# PMMA bone cement with AgNP@CDs nanocomposite for infection control and inflammation mitigation

**DOI:** 10.1093/rb/rbaf086

**Published:** 2025-08-14

**Authors:** Ihsan Ullah, Jian Ju, Yapei Song, Siyi Chen, Mengshi Chen, Siran Wang, Wenzhen Zhang, Wenhui Chen, Zhifeng You, Huaqiong Li, Feng Wen, Wei Zuo

**Affiliations:** Joint Research Centre on Medicine, The Affiliated Xiangshan Hospital of Wenzhou Medical University, Ningbo, Zhejiang 315700, China; College of Chemical Engineering, Fuzhou University, Fuzhou, Fujian 350116, China; Zhejiang Engineering Research Centre for Tissue Repair Materials, Wenzhou Institute, University of Chinese Academy of Sciences, Wenzhou, Zhejiang 325001, China; Zhejiang Engineering Research Centre for Tissue Repair Materials, Wenzhou Institute, University of Chinese Academy of Sciences, Wenzhou, Zhejiang 325001, China; Postgraduate Training Base Alliance of Wenzhou Medical University, Wenzhou, Zhejiang 325000, China; Zhejiang Top-Medical Medical Dressing Co. Ltd, Wenzhou, Zhejiang 325025, China; Key Laboratory of Biomaterials and Biofabrication for Tissue Engineering, Gannan Medical University, Ganzhou, Jiangxi 341000, China; Zhejiang Engineering Research Centre for Tissue Repair Materials, Wenzhou Institute, University of Chinese Academy of Sciences, Wenzhou, Zhejiang 325001, China; The Second Affiliated Hospital and Yuying Children’s Hospital of Wenzhou Medical University, Wenzhou, Zhejiang 325035, China; Yuhuan People’s Hospital, Taizhou, Zhejiang 317600, China; Zhejiang Engineering Research Centre for Tissue Repair Materials, Wenzhou Institute, University of Chinese Academy of Sciences, Wenzhou, Zhejiang 325001, China; Faculty of Biomedical Engineering, Shenzhen University of Advanced Technology, Shenzhen 518107, China; Joint Research Centre on Medicine, The Affiliated Xiangshan Hospital of Wenzhou Medical University, Ningbo, Zhejiang 315700, China; Zhejiang Engineering Research Centre for Tissue Repair Materials, Wenzhou Institute, University of Chinese Academy of Sciences, Wenzhou, Zhejiang 325001, China; Postgraduate Training Base Alliance of Wenzhou Medical University, Wenzhou, Zhejiang 325000, China; Zhejiang Top-Medical Medical Dressing Co. Ltd, Wenzhou, Zhejiang 325025, China; Key Laboratory of Biomaterials and Biofabrication for Tissue Engineering, Gannan Medical University, Ganzhou, Jiangxi 341000, China; Joint Research Centre on Medicine, The Affiliated Xiangshan Hospital of Wenzhou Medical University, Ningbo, Zhejiang 315700, China

**Keywords:** antimicrobial, bacterial infection, nanocomposite, biocompatibility, bone cement

## Abstract

Bioinert poly(methyl methacrylate) (PMMA) is widely employed as a bone cement material in orthopedic and trauma surgery applications; however, its susceptibility to bacterial infection and bioinert nature limits its clinical applications. In this study, we developed a PMMA-based bone cement incorporating a silver nanoparticle-carbon dots (AgNP@CDs) nanocomposite (∼70 nm) at concentrations (2 wt%) with a Young’s modulus (324.74 ± 7.08 MPa) to simultaneously combat bacterial infections, minimize cytotoxicity and support tissue regeneration. The CDs stabilize and functionalize AgNPs, improving their dispersion and bioavailability while enabling the controlled and sustained release of antimicrobial ions through incorporation with bone cement. The antibacterial efficacy of the composite was thoroughly evaluated, revealing its ability to disrupt bacterial cell membranes, generate reactive oxygen species and inhibit bacterial growth. These mechanisms collectively contribute to a significant reduction in bacterial growth of up to ∼90% in both *in vitro* and *in vivo* studies. The incorporation of AgNP@CDs ensures sustained antimicrobial activity, preventing bacterial colonization by controlling the leaching of Ag ions. Biocompatibility assessments showed that the PMMA composite (PMMA@2Ag-CDs) significantly improved cell proliferation, adhesion and migration compared with pure PMMA bone cement. Additionally, histological analysis revealed that the PMMA group showed a fibrous layer thickness of 699 ± 35.32 µm, indicative of inflammation, while the PMMA@2Ag-CDs group reduced this thickness from 301.18 ± 22.42 µm on day 7 to 198.07 ± 15.21 µm on day 14, significantly decreasing inflammation. The PMMA@2Ag-CDs composite demonstrated better tissue integration, with organized collagen deposition and enhanced angiogenesis, indicating more efficient tissue regeneration. The reduced inflammation and improved tissue remodeling suggest that this composite promotes a more favorable tissue regeneration environment and minimizes complications. This study demonstrates that the PMMA@2Ag-CDs composite offers a promising solution for the prevention of infections and mitigation of inflammatory responses. Functionalization of bone cement through the incorporation of Ag nanoparticle-carbon dot nanocomposites is a promising strategy with potential practical applications in orthopedic and trauma surgery.

## Graphical abstract

**Figure:**
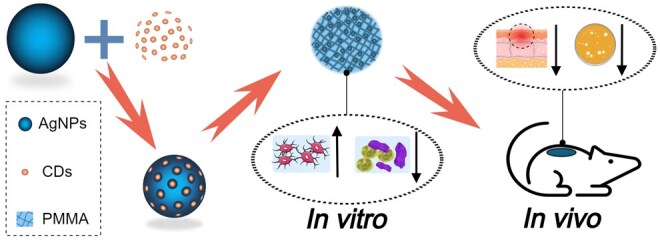

## Introduction

Poly(methyl methacrylate) (PMMA)-based bone cement and its derivatives have been used in orthopedic and trauma surgeries for decades to stabilize and secure artificial joints and implants at defective sites [1, 2]. PMMA serves as a filler that forms a secure space to hold the implant tightly against the bone [3]. It consists of two sterile components: a powder (PMMA and methyl methacrylate (MMA) copolymer) and a liquid (MMA monomer and inhibitor). When mixed, the liquid monomer polymerizes around the PMMA particles, forming hardened PMMA through an exothermic reaction. PMMA is known for its reliability and ease of use in clinical practice and has a proven long survival rate with cemented prostheses. The market for PMMA-based bone cement is expected to grow to $1.62 billion by 2029, with a compound annual growth rate of 6.7% (The Business Research Company). This growth is attributed to its excellent clinical efficacy resulting from the strong mechanical bond of PMMA with implants, ensuring the uniform distribution of implant loads and enhancing structural stability [4]. PMMA is a mechanically strong but biologically inert bone cement. To enhance the biological properties of PMMA, numerous studies have been conducted (Supplementary Table S1), including the incorporation of hydroxyapatite (HA) [5], tricalcium phosphate (TCP) [6], or glassy carbon [7]. Nevertheless, implanted PMMA cement is prone to bacterial infections, mainly due to its moist *in vivo* environment and thermal instability. To enhance the antibacterial ability, numerous additives, including antibiotics such as gentamicin, tobramycin, erythromycin, cefuroxime, vancomycin and colistin, have been incorporated into the powder component of PMMA. The basic requirement for mixable antibiotics is that they should be heat-resistant to high polymerization temperature of PMMA [8] and capable of lasting for a prolonged period to maintain their effectiveness. This transforms PMMA into a drug delivery system that administers the demanded drugs directly to the surgical locations [9]. Antibiotics administered using conventional formulations are not targeted and often transport to undesired sites, resulting in the emergence of resistance and side effects. The dosage administered through bone cement can be significantly lower than that required for conventional systemic injection in clinical practice. Nevertheless, the development of practical, biocompatible and durable antibacterial agents is crucial for preventing the spread of drug-resistant bacteria.

Recently, nanomaterials have emerged as a new alternative therapy for combating drug-resistant bacteria, especially when conventional antibiotics are ineffective [10, 11]. This emerging field of antibacterial research includes various nanomaterials, such as Cu, CaO_2_, ZnO, MoS_2_, graphene oxide and sliver [12–15]. Silver-based (Ag) materials have attracted considerable attention as antimicrobial agents owing to their significant antibacterial properties, especially against drug-resistant infections [16, 17]. Ag nanoparticles (AgNPs) are currently the most widely commercialized nanomaterials for bone grafts, skin wound healing, photocatalysis, etc, due to their broad-spectrum antimicrobial properties [18–20]. However, traditional AgNPs tend to agglomerate easily, have poor dispersion and are quickly oxidized into Ag ions, which limits their antibacterial ability [21]. Additionally, their potential toxicity to cells and the environment raises concerns. The toxicity of AgNPs is mainly associated with their ability to accumulate in various tissues and organs, where they may induce adverse effects such as cell necrosis, apoptosis or genetic mutations [22]. For example, AgNPs deposited in the lungs may cause pneumonia and asthma [23]. Moreover, the large surface area of AgNPs may increase the release of Ag ions, further enhancing their toxic effects. Therefore, appropriate surface passivation or support material is required for AgNPs to preserve their antibacterial properties while addressing these challenges.

Carbon dots (CDs) have recently gained considerable attention as zero-dimensional materials for biomedical applications [24]. These tiny wonders stabilize metal nanomaterials, revolutionize surface functionalization, enhance chelation and reduction of nanomaterials, boost bioavailability, and offer an easy and cost-effective synthesis pathway [24, 25]. By incorporating CDs into AgNPs (AgNP@CDs), CDs can function as both reducing and stabilizing agents in the synthesis of AgNPs, thereby preserving their antibacterial efficacy and diminishing their toxicity [12, 26]. This is due to the oxygen-containing functional groups on the surface of the CDs (such as carbonyl, carboxyl, hydroxyl and epoxy groups), which act as efficient electron donors and acceptors [27, 28]. The immobilization of CDs on AgNPs increases their negative surface charge and hydrophilicity [29]. Moreover, the proximity of the AgNPs quenches the CDs, causing energy transfer from the CDs to the AgNPs through surface plasmon enhancement [30]. This interaction not only optimizes the antibacterial activity of AgNPs but also reduces their cytotoxicity, paving the way for the development of safer and more effective nanomaterials for therapeutic applications.

To address the myriad intricate challenges in clinical practice, various additives have been incorporated into bone cement matrices [31]. However, *in vitro* and *in vivo* studies have shown that only a small amount of these additives are released from bone cement. This is primarily because the release of additives from bone cement is a surface phenomenon [32]. Furthermore, the sustained release of additives from bone cement is significantly influenced by body fluids that permeate the polymer matrix, necessitating moderate porosity within the internal microstructure of cement. Nonetheless, even the addition of a small quantity of additives often results in detrimental effects on the strength of bone cement. The significant impact of impurities on the elastic modulus characteristics of cement has been confirmed in previous studies [33, 34]. Additionally, the mechanical properties of bone cement change significantly with different preparation processes [35]. To meet clinical requirements, it is essential to balance the concentration of additives, the internal structure of the bone cement and the strength requirements of the application site.

In this study, to modulate the toxicity of AgNP@CDs, as evidenced by multiple studies [36, 37], and to apply them in clinical practice scenarios, we developed PMMA-based bone cement that incorporated AgNPs and CDs nanocomposites to simultaneously address the challenges of bacterial infection and cytotoxicity while enhancing tissue regeneration. The incorporation of CDs plays an essential role in stabilizing and functionalizing AgNPs, significantly enhancing their dispersion, bioavailability and antibacterial efficacy through chelation and surface modification. The PMMA matrix, a key component in controlling the leaching of Ag ions from AgNP@CDs, regulates the sustained release of Ag ions while ensuring their targeted delivery, thereby maximizing the antibacterial efficacy and minimizing the potential toxicity of AgNP@CDs. The antibacterial action of the PMMA-containing AgNP@CDs nanocomposite was thoroughly evaluated using multiple techniques, confirming its ability to disrupt bacterial membranes, generate reactive oxygen species ROS and effectively inhibit bacterial growth. Extensive *in vitro* and *in vivo* studies have further validated its synergistic effects, positioning this composite as a promising next-generation material capable of combating bacterial infections and significantly improving tissue regeneration. In contrast to prior research that predominantly concentrated on either antibiotic incorporation or the utilization of metal nanoparticles, this study distinctively integrates non-metallic CDs with AgNPs within a single composite, thereby amalgamating the benefits of both materials. This integration not only augments antimicrobial efficacy but also mitigates the potential cytotoxicity associated with AgNPs and facilitates tissue regeneration. The employment of CDs to stabilize AgNPs and modulate ion release further differentiates this work from other PMMA-based bone cement, offering a more effective, biocompatible and controlled-release system compared to traditional methodologies. This innovative approach addresses critical challenges in infection control, wound healing and combat against antibiotic resistance, presenting a promising and sustainable alternative to conventional bone cement.

## Materials and methods

### Chemical reagents

The citric acid (CA), thiourea (CH_4_N_2_S), hydroxylamine hydrochloride, Triton X-100, sodium hydroxide (NaOH), hydroxylamine hydrochloride (HH) and silver nitrate were purchased from Aladdin Shanghai, China. NaAc-HAc buffer (200 mM, pH 4.26), phosphate-buffered saline (PBS) buffer (100 mM, pH 7.0), Dulbecco’s modified eagle medium (DMEM, 1195), fetal bovine serum (FBS, S9020) were purchased from Beijing Solarbio Science& Technology Co. Ltd (Beijing, China). Bone cement (PMMA) was purchased from Heraeus Medical GmbH (Hanau, Germany). ROS detection kit (S0033S) was obtained from Beyotime Biotechnology Inc (Shanghai, China). Nanopure water (18.3 MΩ cm) was used in all experiments.

### Bone cement preparation, physical and chemical properties characterizations

#### Preparation of CDs

In this study, CDs were synthesized using a hydrothermal method. A 1:2 mass ratio of CH_4_N_2_S and CA was dissolved in DMF and heated to 160°C for 6 h in an autoclave [38]. After allowing the mixture to cool to room temperature, the products were centrifuged at 10 000 rpm for 10 min. Subsequently, 2.5 mL of the supernatant was gradually added to 20 mL of NaOH solution (50 mg/mL). The resulting CDs were stored at a concentration of 2.0 mg/mL in a refrigerator at 4°C for later use.

#### Preparation of AgNPs coated with CDs (AgNP@CDs)

AgNP@CDs were synthesized using an ultrasonic method at room temperature [39]. In summary, 4 mL of a 0.2 mg/mL CDs solution was diluted to a total volume of 44 mL, and 1 mL of a 10 mg/mL AgNO_3_ solution was added to it. Then, 5 mL of a solution containing 0.33 μL of Triton X-100, 15 mM HH and 30 mM NaOH was added to the solution. The initially colorless mixture rapidly darkened, indicating the formation of AgNP@CDs. The solution was sonicated for 30 min, and then the AgNP@CDs were centrifuged twice for 10 min each to remove any unreacted CDs. The final concentration of the solution was adjusted to 2 mg/mL for storage purposes.

#### Fabrication of bone cement and bone cement composite scaffolds

According to the supplier’s instructions, 1 g of bone cement powder (PMMA) was used as the polymeric monomer and was finely ground using a mortar and pestle. Subsequently, 1 mL of MMA was added as an initiator. The mixture of liquid (MMA) and powder (PMMA) using a mortar initiates two distinct processes. In the initial physical mixing process, the polymerization fractions and liquid come into contact with each other individually, forming a viscous fluid. The fluid was transferred into a mold, followed by the initiation of the polymerization reaction, which commenced through the action of free radicals generated by the initiator at room temperature. As the number of free radicals generated increases, polymer molecules begin to form owing to the transition to the form of polymer chain segments.

To form bone cement composite scaffolds, CDs and AgNP@CDs were synthesized using an approach similar to that used for preparing bone cement. Similarly, 1 g of powder (PMMA) was blended with 10 or 20 mg of CDs or AgNP@CDs, which were ground together for 10 min using a mortar and pestle. Subsequently, 1 mL of the initiator was added, mixed quickly and transferred to a mold. The bone cement composites were denoted as PMMA@CDs, PMMA@1Ag-CDs and PMMA@2Ag-CDs, depending on the type of blended particles and the amount of AgNP@CDs used. After the reaction, the sample was removed and stored in an electronically dry cabinet for subsequent use.

#### Characterization of CDs, AgNP@CDs, bone cement and bone cement composite scaffolds

The morphology and size of the CDs and AgNP@CDs were characterized using high-resolution transmission electron microscopy (HRTEM, FEI Talos F200S, China). X-ray photoelectron spectroscopy (XPS, Thermo Scientific K-Alpha, USA) was used to determine the quantitative atomic composition and chemistry of the synthesized CDs and AgNP@CDs. UV–Vis absorption spectra were obtained to determine the characteristic absorption peaks of CDs and AgNP@CDs using a TU-1901 spectrometer (Beijing, China). A fluorescence microscope (Axio, Zeiss, Germany) was used to detect fluorescence signals from the stained cells. Element in bone cement composites, cell and bacteria morphology were examined by scanning electron microscope (SEM, Phenom Pharos, Netherlands). The mechanical properties of bone cement and bone cement composite scaffolds were assessed as previously reported with some modifications [8] using a universal testing system (Instron 5594; Instron, Norwood, MA, USA). After the load cell was calibrated, the specimens were compressed at a rate of 10 mm/min. The results were recorded, and the Young’s moduli were compiled accordingly. The test was repeated at least five times for each specimen set.

### Biocompatibility and antibacterial activity characterizations

#### Cell proliferation

The potential toxicity of PMMA, PMMA-containing CDs and AgNP@CDs was evaluated using a cell counting kit-8 (CCK-8, Beyotime, China). After three passages, fibroblasts (L929) and osteoblast precursor cells (MC3T3) were used in this study. All PMMA disks were washed with PBS and sterilized before use. Sterilized disks were incubated with DMEM for 24 h at 37°C. The conditioned media from the incubation was collected and supplemented with 10% FBS and 1% antibiotics. L929 cells were plated into 96-well plates at a density of 0.5 × 10^4^ cells per well and incubated for 24 h in a humidified incubator (5% CO_2_). Cytotoxicity was assessed at 1, 3 and 5 days by adding 10% CCK-8 solution to each well, followed by a 3-h incubation in the dark. The optical density (OD) was recorded at 450 nm using a microplate reader, where the OD values served as indicators of cell viability and toxicity of the PMMA composites.

For live/dead staining, cells were cultured with conditioned media for 3 days, followed by a brief wash with PBS. The cells were then incubated with calcein-AM/PI solution for 30 min in the dark. After staining, the cells were washed with PBS to remove any excess dye. The stained cells were observed using a fluorescence microscope, allowing for clear differentiation between live and dead cells [40]. This approach provided valuable information on the cytotoxic effects of PMMA composites.

#### Cell adhesion

The morphology of the seeded L929 cells on the surfaces of the various PMMA disks was further analysed using scanning electron microscopy. After 72 h of incubation, the samples were washed thrice with PBS and fixed with 2.5% glutaraldehyde. Following fixation, the disks were rinsed thrice with PBS. The disks were then dehydrated using a graded series of ethanol concentrations (10%, 30%, 50%, 70%, 70%, 85%, 85%, 90% and 100%) and subsequently dried. Before SEM, all the disks were coated with platinum via sputtering.

#### Antibacterial activity study

The antibacterial performance of PMMA and PMMA composites was examined against *Staphylococcus aureus* (*S. aureus*) and *Escherichia coli* (*E. coli*). Prior to the experiment, both bacterial strains were cultivated in Luria–Bertani (LB) medium, as described in a previous study [41]. For the disk diffusion method, 100 μL of a bacterial suspension (10^7^ CFU/mL) was inoculated onto an agar plate. Sterilized circular disks (8 mm diameter) were placed on the inoculated plates and incubated for 24 h at 37°C. The antibacterial efficacy of the PMMA and PMMA composites was determined by measuring the size of the inhibition zone around the samples.

To determine the minimum inhibitory concentration (MIC) of the samples, *S. aureus* and *E. coli* were further diluted to 10^5^ CFU/mL. Subsequently, 100 μL of the diluted bacterial solution was added to each sample in 48-well plates. The samples were cultured under appropriate conditions, and turbidity of the medium was assessed using digital imaging [13]. The antibacterial ratio was calculated using Equation (1)


(1) K=(C−E)/C×100,

where *C* represents the OD of the control group and *E* represents the OD of the experimental group.

For SEM analysis of the bacteria, the sample preparation procedure was the same as that for the SEM analysis of the cells. For the ROS test, bacterial cultures were exposed to PMMA and PMMA-containing CDs and AgNP@CDs nanocomposite for 12 h. ROS production was assessed by adding a fluorescent probe DCFDA to the bacterial suspension after exposure, followed by incubation for 30 min at 37°C. Fluorescence intensity was measured using a microplate reader at 485 nm excitation and 530 nm emission wavelengths.

### Subcutaneous implant-related infection model set up and assessments of antibacterial activity and inflammation

*In vivo* antibacterial activity and inflammatory reactions were assessed via subcutaneous implantation according to previous report [42] with some modifications. In brief, all procedures followed the guidelines set by the Animal Care Committee of the Wenzhou Institute, University of Chinese Academy of Sciences. Before the experiments, Sprague Dawley rats (300–400 g) were acclimatized to their new environment. The rats were randomly assigned to two groups: PMMA and PMMA@2Ag-CDs. After anesthesia with pentobarbital sodium, the rat’s fur was shaved, and their skin was cleaned using povidone-iodine. A 3 × 5 cm^2^ incision was made along the spine, and the samples pre-incubated with *S. aureus* (100 μL, 1 × 10^5^ CFU/mL) for 1 h were implanted subcutaneously into rats. The rats were euthanized after 7 and 14 days, and the implants were retrieved from the rats. The implants were sonicated in PBS to remove any remaining bacteria, and the antibacterial efficacy was assessed using the plate counting method. Additionally, the surrounding soft tissues were fixed in 10% buffered formalin, dehydrated with graded alcohol, embedded in paraffin, sectioned and stained with hematoxylin and eosin (H&E) and Masson’s trichrome stains. Immunohistochemistry for CD31 was performed to evaluate the inflammatory responses.

### Statistical analysis

All biological experiments were conducted in triplicate, and the results are expressed as the mean ± standard deviation. Statistical significance was assessed using a one-way ANOVA test. *P-*values of less than 0.05 were considered statistically significant, while *P*-values of less than 0.01 or 0.001 were regarded as highly significant.

## Results and discussion

### Physical and chemical properties characterization

#### Characterization of CDs and AgNP@CDs

**Figure 1.**
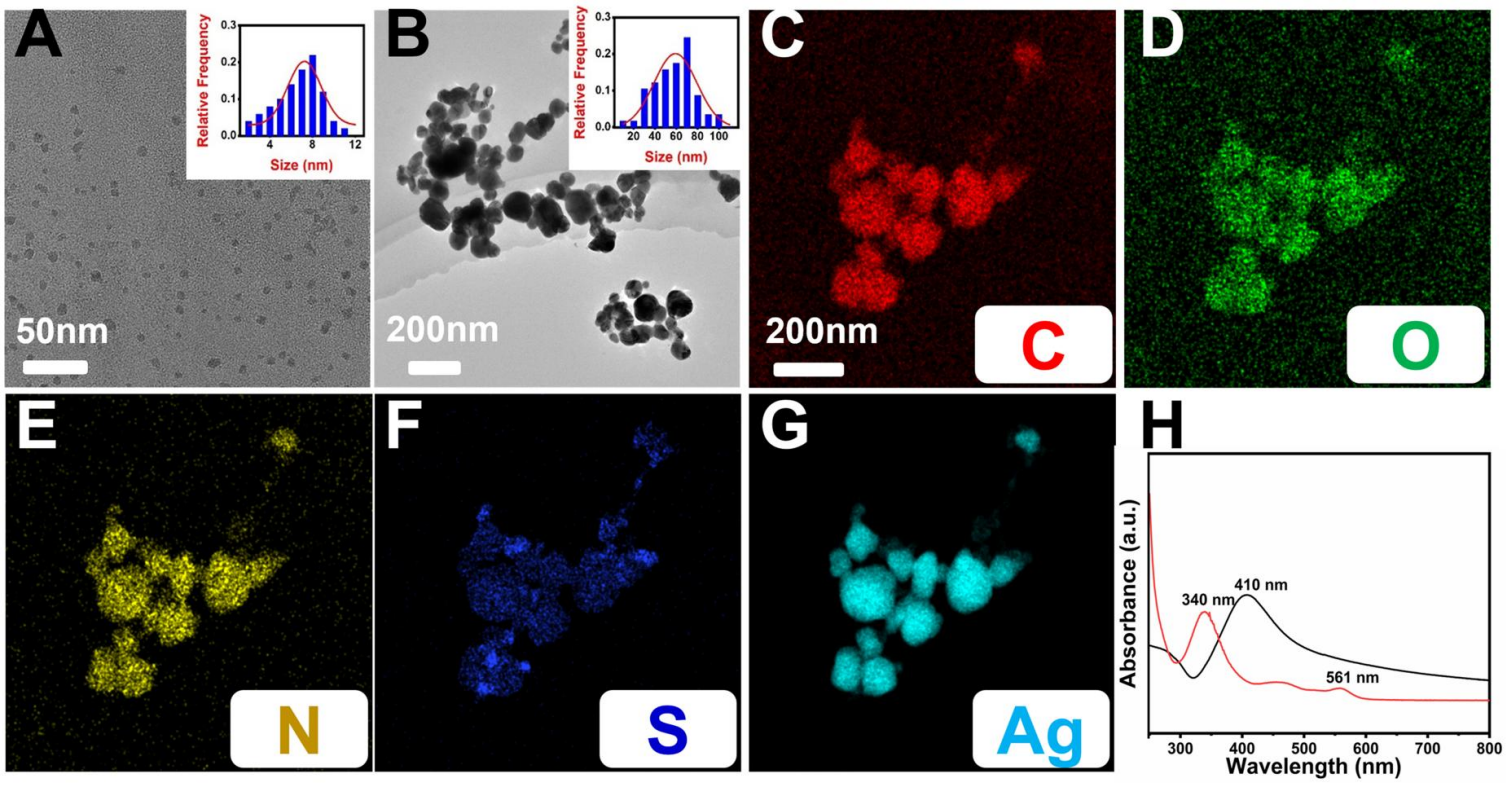
(**A**) TEM images of CDs and insert showing the histograms. (**B**) AgNP@CDs, inset showing the size histograms of the AgNP@CDs. EDX elemental mapping images for C (**C**), O (**D**), N (**E**), S (**F**) and Ag (**G**) of AgNP@CDs and (**H**) UV–vis absorption spectra of CDs (red line) and AgNP@CDs (black line) in water.

The TEM morphologies of the CDs and AgNP@CDs were examined (Figure 1). The CDs exhibited a small schistose morphology with a highly crystalline structure, as shown in Figure 1A. The size histograms presented in the insert of Figure 1A indicate that the average diameter of the CDs is approximately 8 ± 0.5 nm. The AgNPs wrapped with CDs (AgNP@CDs) have a well-defined morphology with an average diameter of 70 ± 5 nm (Figure 1B and insert). The nanocrystal information of the AgNP@CDs was also analysed using scanning TEM (STEM) coupled with energy-dispersive X-ray spectroscopy (EDX) (Figure 1C–G) The EDX image of AgNP@CDs shows a clear separation of the C, N, O and S elements from the CDs, which are evenly distributed over the AgNPs. This confirmed that the CDs were successfully coated onto the surface of the AgNPs. The absorption spectra (UV–visible) of the aqueous dispersions of the CDs (red) and AgNP@CDs (black) are shown in Figure 1H. Characteristic absorption spectral peaks at 340 and 560 nm result from n–π* leaps of C=N/C=O. C–N and C–S enriched on the surface of the CDs. In comparison to CDs, whose UV–vis absorption spectrum showed a sharp and strong peak at approximately 410 nm, indicating the successful synthesis of AgNPs [43]. Notably, the characteristic absorption peaks of CDs at 340 and 561 nm were not observed in AgNP@CDs, presumably because the CDs were tightly adhered to the AgNPs surface and created a shell–core structure.

**Figure 2.**
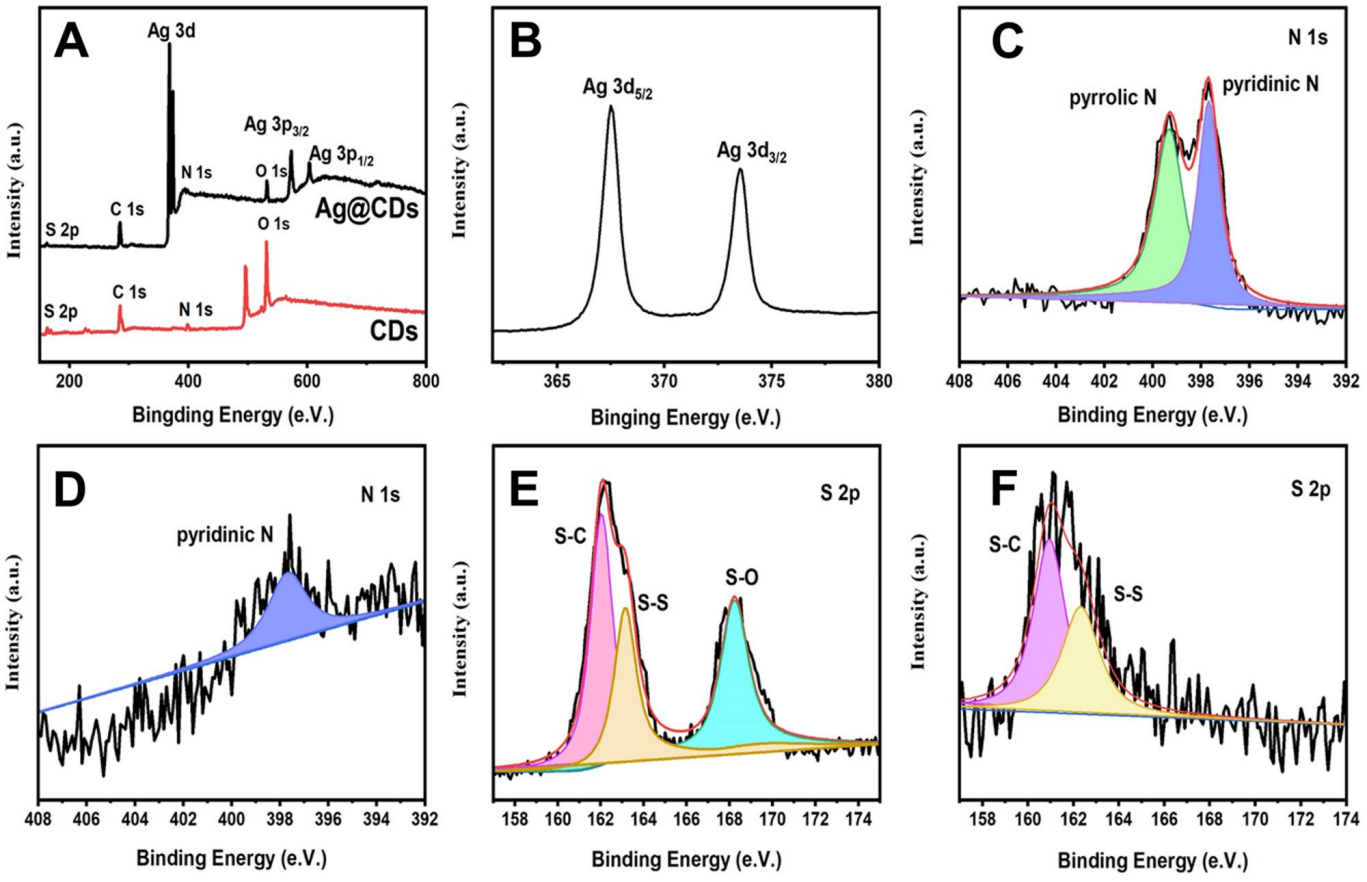
(**A**) XPS spectra of the synthesized CDs and AgNP@CDs, (**B**) Ag 3d, (**C**) N 1s of CDs, (**D**) N 1s of AgNP@CDs, (**E**) S 2p of CDs and (**F**) S 2p of AgNP@CDs.

Figure 2 shows the XPS spectra of CDs and AgNP@CDs with C 1s as the reference. The characteristic XPS peaks of AgNPs and CDs are shown in Figure 2A. Figure 2B shows the high-resolution XPS Ag 3d spectrum, which contains two prominent peaks at 367.6 and 373.6 eV, which can be attributed to the binding energies of Ag 3d_5/2_ and Ag 3d_3/2_, respectively. This result indicates a strong presence of metallic Ag. In the XPS N 1s spectrum (Figure 2C), the peaks at 399.2 and 400.5 eV are attributed to pyridinic N and pyrrolic N, respectively. In contrast, in Figure 2D, only the peak of pyridinic N is found in AgNP@CDs, while the more electron-donating pyrrolic N disappears due to its involvement in the Ag preparation process. A similar phenomenon was observed for the S 2p spectrum of CDs (Figure 2E) and AgNP@CDs (Figure 2F), where the S–O peak at 168.3 eV disappeared in the spectrum of AgNP@CDs. The results indicate that the successful preparation of silver nanoparticles and CDs binding with AgNPs was accomplished mainly by S, N and O in the CDs.

#### Morphology of the PMMA composites

**Figure 3.**
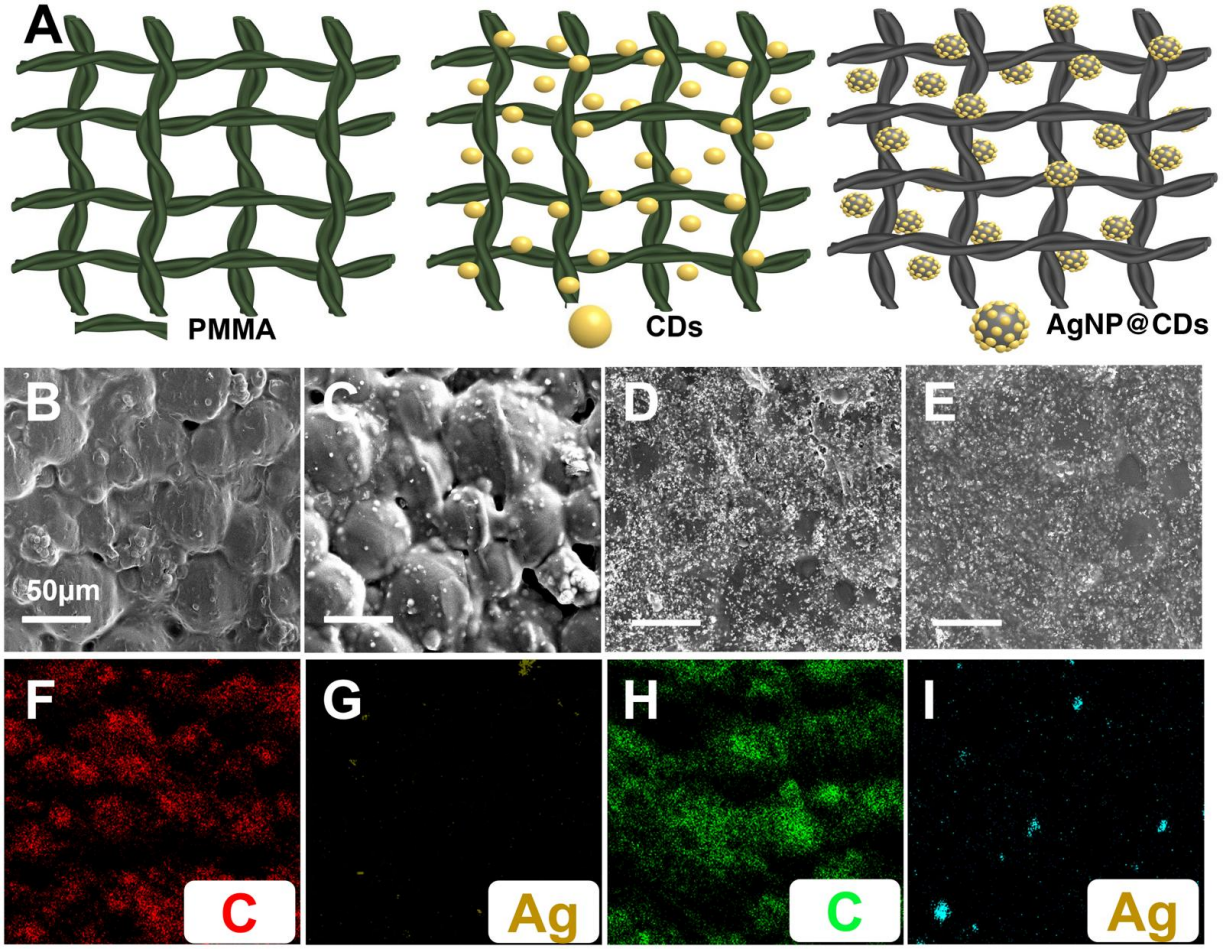
(**A**) Schematic representation of the structure of PMMA, containing CDs and AgNP@CDs nanocomposites, (**B**) SEM morphology of the PMMA surface, (**C**) PMMA-containing CDs, (**D**) PMMA@1Ag-CDs, (**E**) PMMA@2Ag-CDs, (**F** and **G**) mapping of PMMA@1Ag-CDs, and (**H** and **I**) EDX mapping of PMMA@2Ag-CDs showing the existence of carbon and Ag.

Figure 3A shows a schematic of how the CDs and AgNP@CDs nanocomposite were incorporated into the PMMA structure. Figure 3B shows the SEM image of the pure PMMA surface, which exhibits a smooth and relatively uniform texture without any distinct spherical shapes. Figure 3C depicts PMMA incorporated with CDs, showing that the morphology of the PMMA surface remains unaffected by the incorporation of CDs. The CDs were visible on the PMMA surface, indicating their successful dispersion without altering the surface texture, and the PMMA spheres were tightly attached during polymerization. In contrast, the PMMA incorporated with the AgNP@CDs nanocomposite covered the PMMA spheres homogeneously, and the nanocomposite filled the gaps between the boundaries of the PMMA spheres (Figure 3D and E). The uniform distribution of the nanocomposite confirmed its stable dispersion on the PMMA surface. The EDX mapping further confirms the homogeneous distribution of carbon and Ag on the PMMA matrix in (Figure 3F–I).

#### Mechanical property of the PMMA composites

Uniaxial compression tests were performed to assess the mechanical properties of bone cement and bone cement composites. The results (Supplementary Figure S1) demonstrated that the Young’s modulus of PMMA@2Ag-CDs was lower than that of PMMA, consistent with the findings of a previous study [31]. Nevertheless, no significant difference was detected between PMMA and PMMA@2Ag-CDs.

#### Silver ion release

**Figure 4.**
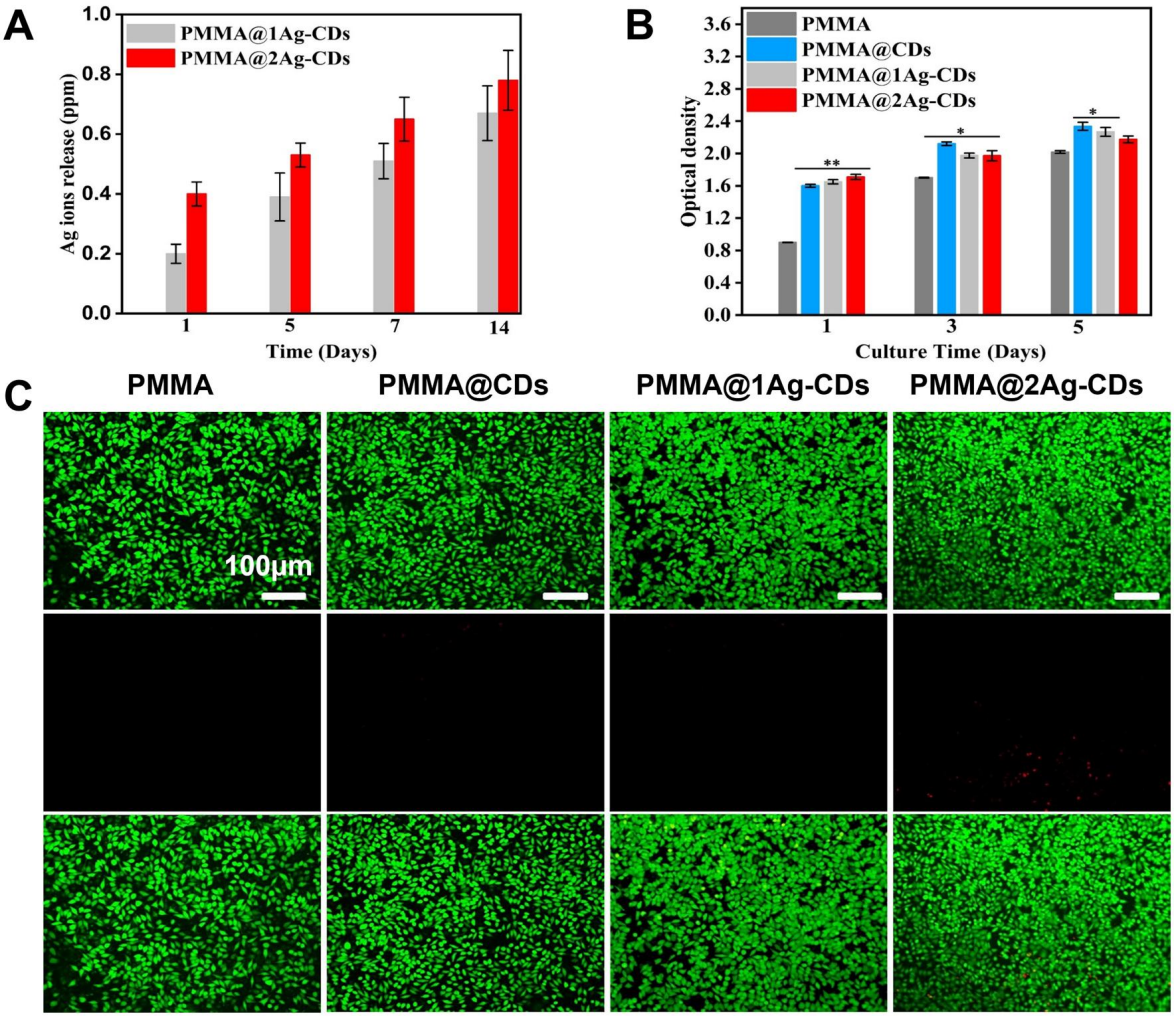
(**A**) Ag ion release of PMMA@1Ag-CDs and PMMA@2Ag-CDs after incubation in cell culture medium, (**B**) cell proliferation of L929 cells and (**C**) live/dead staining of the L929 cells after 72 h of culture.

To determine the Ag ions released, the prepared samples were incubated in the cell culture medium, the extract was collected after 1, 3 5 and 7 days and the ions released were measured via ICP-MS (Figure 4A). The release of Ag ions from the PMMA composites exhibited a time-dependent increase in the cell culture medium. No burst release was observed from any of the samples, and the release was sustained throughout the experiment. PMMA@2Ag-CDs exhibited a higher release rate, indicating that a higher concentration of Ag enhances the availability of Ag ions. After 7 days of immersion, the Ag ions released from PMMA@2Ag-CDs reached 0.72 ppm, which is well below the toxicity threshold [44–48]. The incorporation of CDs significantly enhanced the stability of AgNPs by preventing agglomeration and facilitating a more controlled release of Ag ions. The CDs shell also enhanced the interaction between the Ag nanoparticles and the PMMA matrix, potentially increasing the dispersion of nanoparticles within the polymer. This interaction promotes a more uniform and gradual release of Ag ions from the nanocomposite. The CDs also provide a robust matrix for the AgNPs, enhancing their structural integrity and prolonging their functional lifespan within the composite.

### Biological property characterization

#### Biocompatibility and antibacterial activity *in vitro*

In the healthcare domain, the biocompatibility of materials is crucial. Therefore, we assessed the toxicity of PMMA and various PMMA composites (Figure 4B). L929 cell proliferation exhibited an increasing trend for all samples as a function of time. The cell proliferation of all PMMA composites was significantly higher than that of pure PMMA after 1 and 3 days. Previous studies have shown that CDs induce cell proliferation at optimized concentrations [49]. However, the cell proliferation of pure PMMA increased and became nearly comparable to that of PMMA@2Ag-CDs by the end of the experiment period. This may be due to the effect of AgNPs on metabolic activity of cells. In addition to L929 cells, MC3T3 cells exhibited comparable trends (Supplementary Figure S2). The live/dead staining results were consistent with the CCK-8 findings (Figure 4C). After 72 h of incubation, all the sample surfaces exhibited good cell viability. The excellent cell viability observed in the live/dead staining assay supports the suitability of these composites for long-term biomedical applications, in which sustained biocompatibility is essential. This synergy between CDs and AgNPs nanocomposites stabilizes and uniformly disperses Ag ions within the PMMA matrix and stimulates the proliferation of cells.

**Figure 5.**
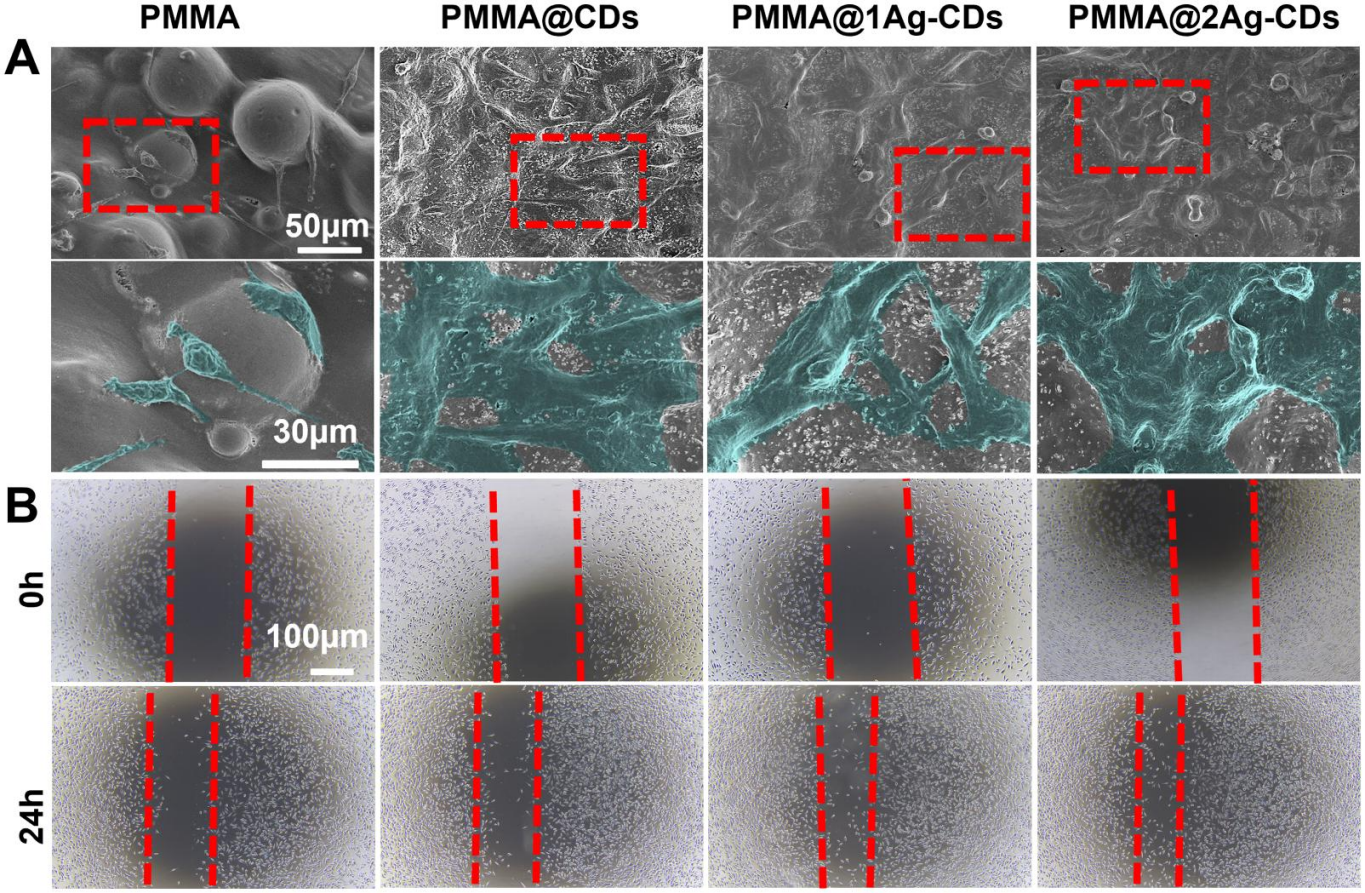
Cell attachment and migration. (**A**) L929 cell SEM morphology (colored in magnified images) after 72 h of incubation on pure PMMA and its various composites and (**B**) cell migration.

Furthermore, cell–material interactions were examined via SEM after 72 h of seeding, revealing distinct differences across the samples (Figure 5A). Pure PMMA revealed poor cell adhesion with sparse and unevenly distributed cells owing to its inert nature [50]. The inclusion of CDs in PMMA surfaces enhances cell spreading with extended projections, such as filopodia and lamellipodia, promoting strong cell adhesion. Even with the introduction of Ag in PMMA@1Ag-CDs and PMMA@2Ag-CDs, cell spreading improved, although some cells appeared more rounded at higher Ag concentrations.

Cell proliferation and migration are essential for rapid re-epithelialization and wound healing, playing a vital role in the recovery process for various types of wounds [51, 52]. Therefore, a migration assay was performed to evaluate cell migration over 24 h. The incorporation of CDs into PMMA (PMMA@CDs) enhanced surface hydrophilicity and promoted protein adsorption, facilitating better cell attachment (Figure 5A) and motility of the cells (Figure 5B). However, cell attachment on substrate was deteriorated after AgNPs was introduced (PMMA@1Ag-CD and PMMA@2Ag-CD). There are some round objects which might be cells without well attachment. Furthermore, research has revealed that CDs could positively impact cell migration due to their significant dose-dependent free radical scavenging activity [53]. Citric acid-based CDs possess antioxidant properties, which scavenge ROS and prevent oxidative stress in cells, thereby reducing inflammation [54]. These findings highlight the combined benefits of the CDs and AgNPs nanocomposite in promoting tissue regeneration while achieving an optimal balance between biocompatibility and antimicrobial ability.

**Figure 6.**
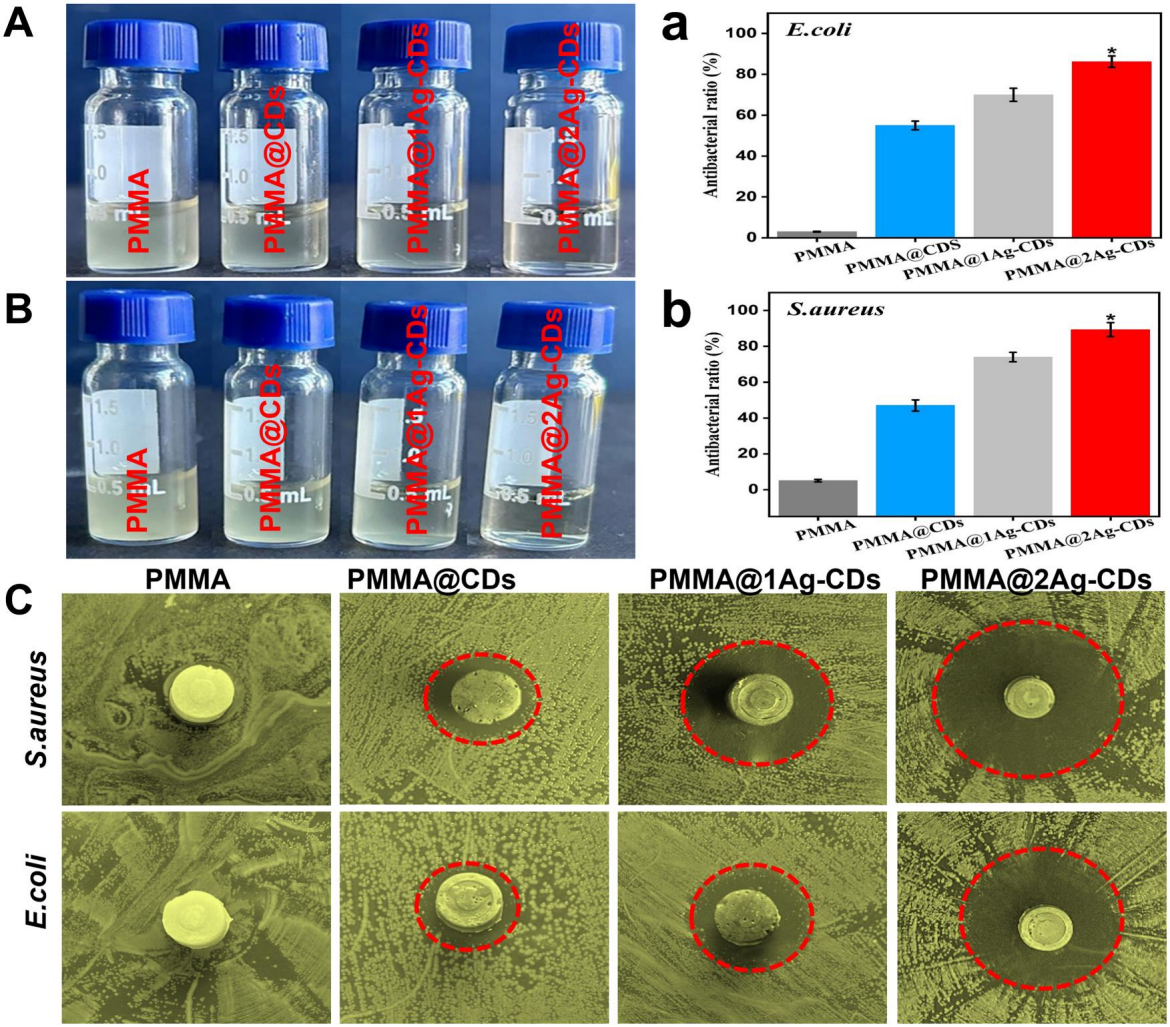
Photographs of the bacterial cultured suspension after incubation of different PMMA composites after exposure to *E. coli* (**A**) and *S. aureus* (**B**), (**a**, **b**) show the quantitative antibacterial ratio of each material against *E. coli* and *S. aureus*, and (**C**) inhibition zone around the various PMMA composite indicating the antibacterial effect against *S. aureus* and *E. Coli*. The circles highlight the inhabited areas.

The antibacterial ability of PMMA and its composites was calculated using LB medium and the disk diffusion method (Figure 6). In addition to their good biocompatibility, PMMA and its composites demonstrated significant antibacterial activity against *S. aureus* and *E. coli*. The LB medium test showed that the addition of CDs and nanocomposites significantly suppressed bacterial growth. Among all the samples, PMMA@2Ag-CDs showed the most pronounced antibacterial effect (transparent medium), achieving 86.2 ± 2.8 inhibition against *E. coli* and 89.3 ± 3.9% against *S. aureus* (Figure 6A and a). This enhanced activity was likely due to the synergistic effects of Ag ions and CDs, where Ag ions disrupt bacterial cell membranes and CDs stabilize AgNPs for prolonged antibacterial action.

*S. aureus*, a common cause of implant infections, can colonize the implant site within 30 min of implantation, may encourage the adhesion and colonization of other bacteria, and contribute to peri-implant lesions [36]. The disk diffusion results further support the importance of targeting *S. aureus* in preventing such infections, as PMMA@2Ag-CDs exhibited the most significant inhibition against *S. aureus* 23.10 ± 1.90 and *E. coli* 19.49 ± 1.61 mm, followed by PMMA@1Ag-CDs and PMMA@CDs (Figure 6C and Supplementary Figure S3), suggesting that gram-positive bacteria, such as *S. aureus*, are more susceptible to the combined effects of AgNPs and CDs, which could help reduce the risk of peri-implant complications.

**Figure 7.**
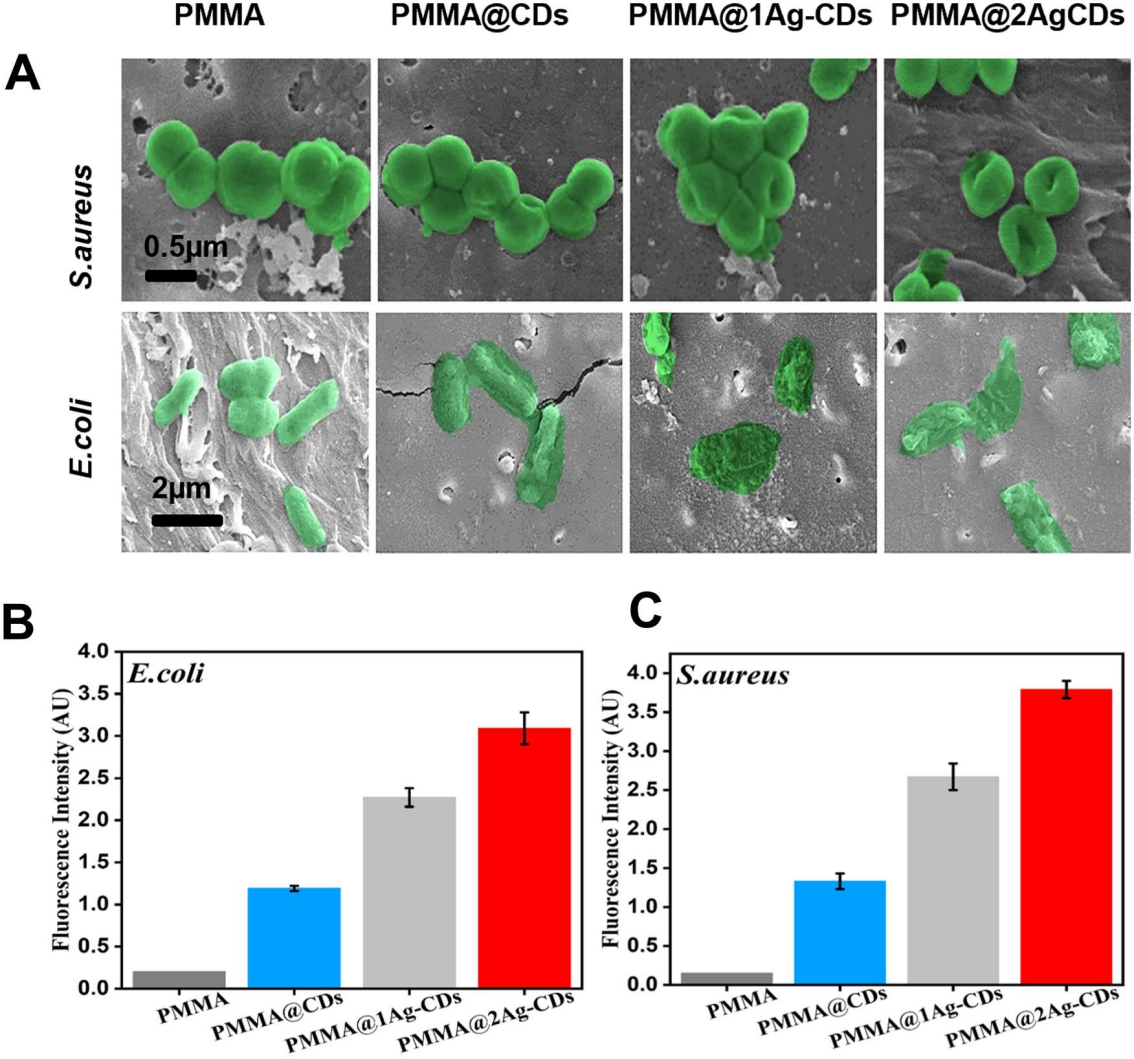
(**A**) SEM morphology of *S. aureus* and *E. coli* on the PMMA and various PMMA composite surfaces, and (**B**) intracellular ROS levels in *E. coli* and (**C**) *S. aureus* cultured on the PMMA and various PMMA composite surfaces.

The SEM morphology and intracellular ROS levels in bacteria provide insights into the antibacterial mechanisms of the PMMA-containing nanocomposite (Figure 7). SEM morphology revealed significant damage to *S. aureus* and *E. coli*, with clear signs of ruptured cell membranes, shape distortion and complete cell lysis, especially on the PMMA@2Ag-CDs surface (Figure 7A). Because AgNP@CDs penetrate the cell walls of the bacteria, they induce permeability and initiate the disintegration of cell membranes through electrostatic interactions [55, 56]. The intracellular ROS levels (Figure 7B) further supported this, with PMMA@2Ag-CDs producing intense green fluorescence, indicating substantial ROS production. ROS induce oxidative stress, damaging bacterial cell membranes, proteins and DNA, leading to cell death [57]. The synergistic effect of Ag ions and CDs drives the enhanced antibacterial activity of PMMA. These results reveal that AgNPs and CDs impose mechanical and physical damage to the bacterial cell wall or membrane, eventually leading to the release of cytoplasmic components and the death of the bacteria [58, 59]. Ag ions and CDs disrupt bacterial membranes and generate ROS. CDs act as electron donors, amplifying ROS production and intensifying the oxidative damage. It is possible to hypothesize that when *S. aureus is* incubated with AgNP@CDs containing PMMA, it produces a large quantity of ROS, which is one of the main reasons for the higher antibacterial activity. Previous studies have also predicted that CDs enhance ROS production efficiency after hybridization [12, 60]. Therefore, the positively charged AgNP@CDs readily accumulated on the bacterial surface, producing higher ROS due to the bacteria penetration and inducing the bacterial cells intercellular ROS, ultimately leading to death [61]. The CDs and PMMA dressing also stabilized AgNPs, ensuring a sustained release of Ag ions (Figure 4A), which prolonged the antibacterial effect.

In contrast, pure PMMA and PMMA@CDs exhibited minimal antibacterial activity, highlighting the essential role of Ag in enhancing antimicrobial properties. These results show that incorporating Ag significantly enhanced the antibacterial efficacy of PMMA composites through membrane disruption, ROS generation and sustained Ag release, making them promising for wound-healing applications. The synergy between AgNPs and CDs enhances the antibacterial effects, offering substantial potential for the development of long-lasting antimicrobial materials.

#### Biocompatibility and antibacterial activity *in vivo*

**Figure 8.**
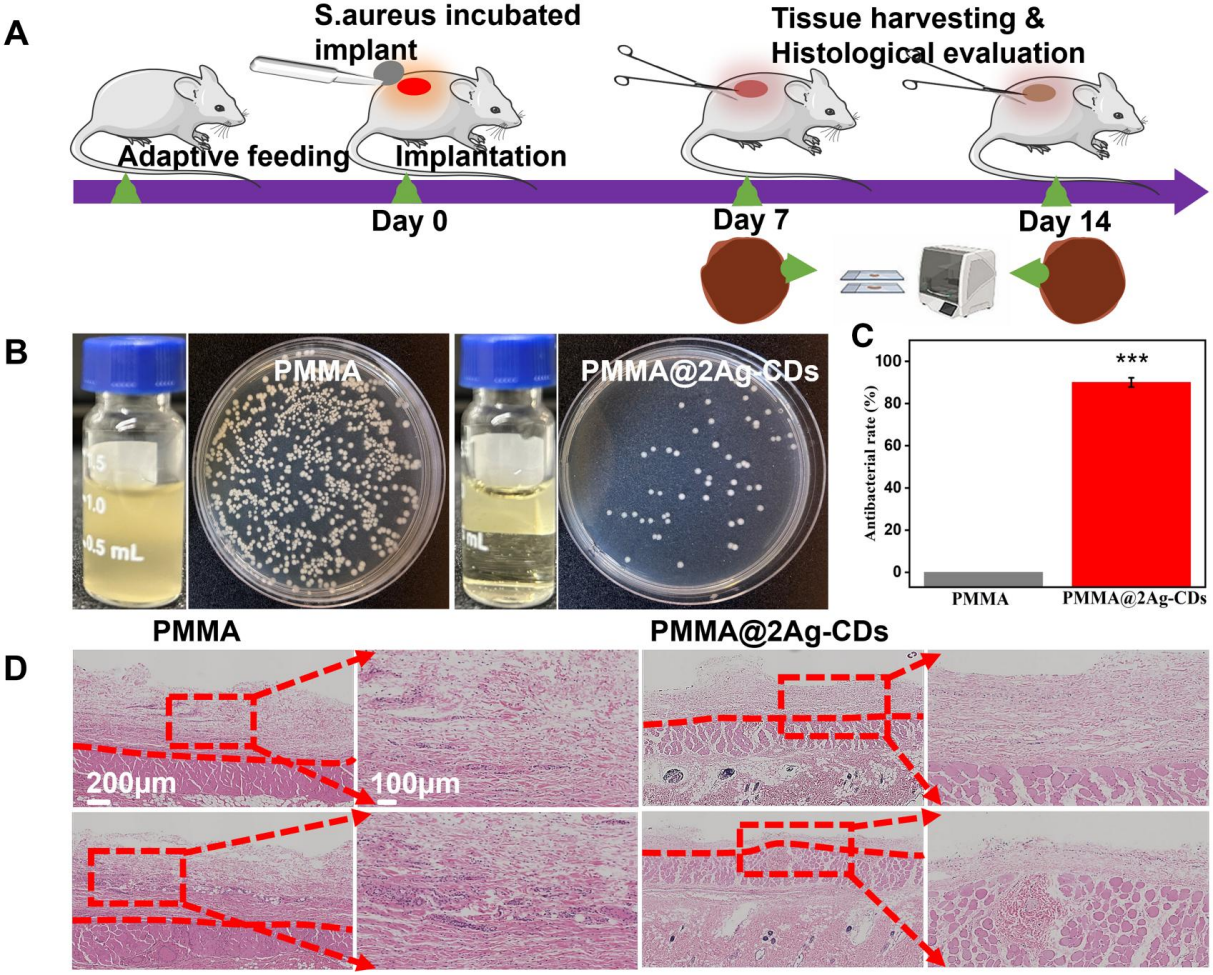
Antibacterial effect and tissue response of PMMA and PMMA@2Ag-CDs implants assessed using H&E staining. (**A**) Experimental setup, with tissue harvesting and histological evaluation at days 7 and 14, (**B**) images of the bacteria-cultured media and plates of the PMMA and PMMA@2Ag-CD_S_ group samples after 14 days of implantation and (**C**) the quantitative antibacterial ratio of each material against *S. aureus*. (**D**) H&E staining of tissue surround subcutaneous implants after 7 and 14 days of implantation.

Based on the *in vitro* results, pure PMMA and PMMA@2Ag-CDs were selected for further *in vivo* experiments. The entire process, including model construction and implant collection, is shown in Figure 8A. Both implants were cultured with *S. aureus* and subsequently introduced into dorsal skin incisions for *in vivo* testing. The implants were retrieved for *in vivo* assessment at specific time points. The antibacterial results are depicted in Figure 8B, where the LB medium cultured with the PMMA implant exhibited turbidity, indicating bacterial growth, whereas the medium cultured with PMMA@2Ag-CDs remained clear and transparent, demonstrating effective antibacterial activity. The plate counting method revealed an *in vivo* antibacterial ability of 90.3 ± 2.5% for PMMA@2Ag-CDs (Figure 8B and C).

Furthermore, to assess the *in vivo* tissue response, peri-implant tissues were collected and analysed using H&E staining. The PMMA revealed a thick fibrous layer 699 ± 35.32 μm around the implant, indicative of a strong inflammatory response and neutrophil infiltration (Figure 8D and Supplementary Figure S4). Many neutrophils were present in the PMMA implant, suggesting an acute inflammatory response to bacterial infection and implant placement. In contrast, the PMMA@2Ag-CDs group exhibited a significantly thinner fibrous layer, indicating a reduced inflammatory response. Notably, the thickness of the fibrous layer in the PMMA@2Ag-CDs group decreased from 301.18 ± 22.42 µm on day 7 to 198.07 ± 15.21 µm on day 14, indicating a continuous amelioration of inflammation over time. The number of neutrophils in the PMMA@2Ag-CDs group was lower than that in the PMMA group, suggesting that the antibacterial properties of the implant effectively controlled the infection and reduced acute inflammation. The prolonged existence of microbes in wound areas can intensify inflammation and postpone the healing period [62]. These findings demonstrate the higher antibacterial and anti-inflammatory properties of PMMA@2Ag-CDs, effectively inhibiting bacterial growth and promoting better tissue healing by reducing inflammation and supporting tissue regeneration.

**Figure 9.**
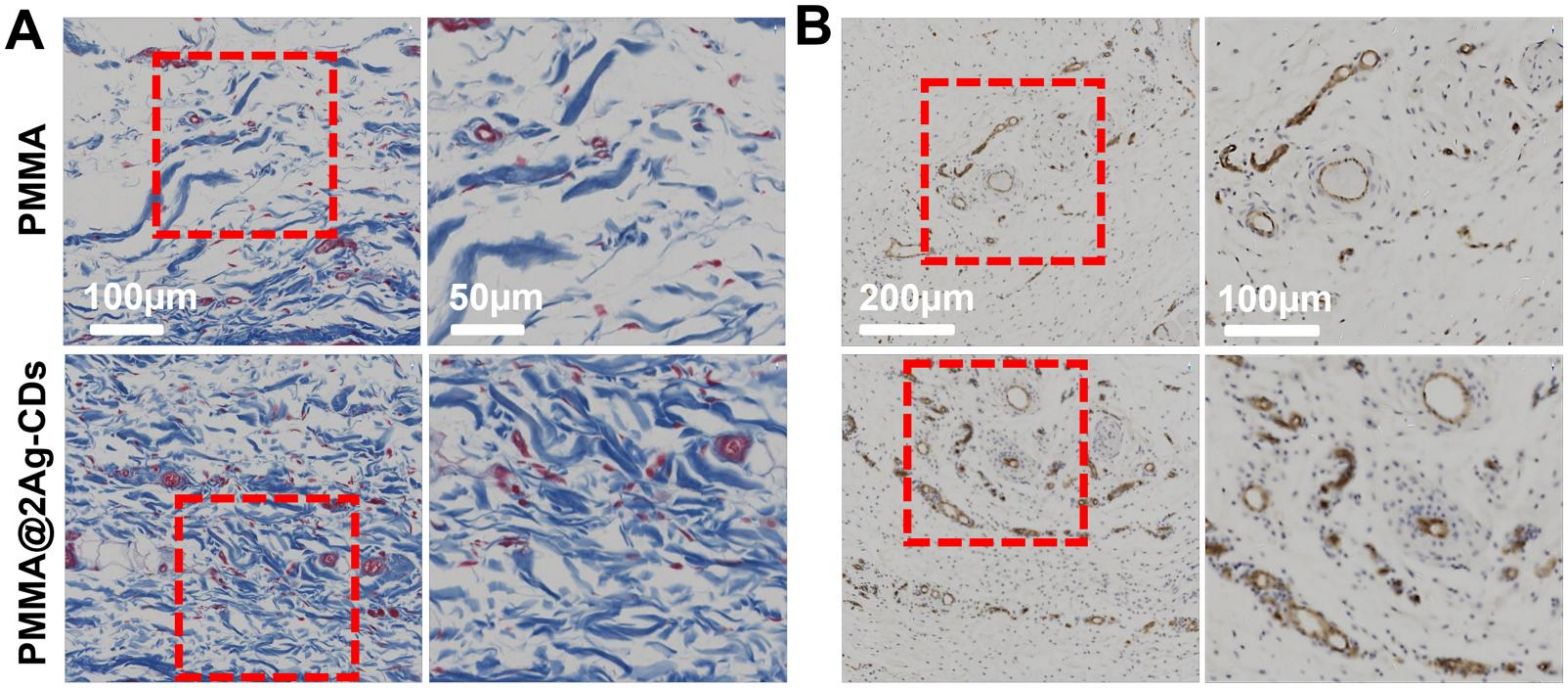
(**A**) Representative images of Masson’s trichrome staining of the PMMA and PMMA@2Ag-CDs implants and (**B**) representative immunohistochemistry staining images of CD31 after 14 days of implantation.

Once an implant is in place, the body naturally forms a capsule of tissue around it, which consists of fibroblasts and collagen fibers. Collagen fibers, primarily secreted by fibroblasts, play a key role in tissue regeneration [63]. Thus, the abundance and viability of fibroblasts on the abutment surfaces are crucial for forming healthy peri-implant biological seals. Therefore, collagen deposition was further assessed using Masson’s trichrome staining due to the disruption of tissue structure and collagen organization caused by bacterial infection and inflammation (Figure 9A). In the PMMA implant, collagen fibers appeared disorganized, indicative of a higher level of inflammation, likely driven by bacterial infection. In contrast, the tissue around the PMMA@2Ag-CDs implant demonstrated more organized collagen deposition, reflecting a reduced inflammatory response and better tissue remodeling due to the antibacterial ability of the AgNPs and CDs nanocomposite incorporated into the PMMA matrix. These results are consistent with those of previous studies showing that Ag-based composites can control inflammation and enhance tissue repair by reducing the microbial burden and promoting organized tissue healing [64, 65]. Regeneration and remodeling of the blood supply system are critical for effective tissue repair. Newly formed blood vessels provide essential nutrients and oxygen for tissue reconstruction and facilitate the removal of metabolic waste products [66]. CD31 staining was used to assess angiogenesis. As shown in Figure 9B, PMMA@2Ag-CDs implants promoted significantly better angiogenesis than the PMMA group. The increased density of vessels and more organized vascular network around the PMMA@2Ag-CDs implants suggest improved tissue perfusion, which is vigorous for efficient tissue regeneration. Angiogenesis, the formation of new vessels, is a key process in tissue regeneration, ensuring adequate oxygen and nutrient supply to injured tissue while supporting immune cell infiltration and collagen deposition. The enhanced vascularization observed in the PMMA@2Ag-CDs group indicates that the antimicrobial properties of the implant reduce bacterial infection and promote vascular remodeling, leading to more efficient tissue regeneration.

The limitation of this *in vivo* study is the absence of an assessment of the long-term stability of the material and the long-term safety of AgNPs. In the long term, the PMMA-based cement incorporating the AgNP@CDs nanocomposite is likely to maintain its antimicrobial efficacy and support tissue regeneration. The intrinsic non-degradable nature of PMMA confirms its sustained mechanical stability and excellent biocompatibility with the surrounding tissues, providing continuous support. The AgNP@CDs composite facilitates controlled Ag ion leaching, remaining within a non-toxic concentration range, and effectively prevents bacterial infections at the implant site. Ag ions disrupt bacterial cell membranes, generate ROS and inhibit bacterial growth while minimizing the detrimental effects on tissue healing. The CDs stabilized and dispersed the AgNPs, enhancing their bioavailability and ensuring long-term antimicrobial activity. Additionally, the composite promotes favorable tissue regeneration by reducing inflammation and encouraging tissue integration, as evidenced by improved collagen deposition and angiogenesis. Overall, the PMMA@2Ag-CDs is expected to provide continuous infection control, support tissue repair and maintain structural integrity beyond 14 days, offering a dual benefit of infection prevention and tissue regeneration, making it a promising alternative to traditional PMMA implants in long-term infection-prone environments.

## Conclusions

In this study, we successfully synthesized and characterized CDs and AgNP@CDs nanocomposites and incorporated them into PMMA-based bone cement at 1 and 2 wt% concentrations to improve their biocompatibility and antimicrobial properties. The incorporation of CDs played a key role in enhancing AgNPs dispersion and controlling ion release, reducing cytotoxicity while maintaining prolonged antimicrobial activity. Furthermore, *in vitro* studies demonstrated that the nanocomposite supported cell proliferation, adhesion and migration, indicating its potential to promote tissue integration *in vivo*. Additionally, antibacterial assessments confirmed that PMMA@2Ag-CDs effectively inhibited bacterial growth by disrupting bacterial membranes and ROS. *In vivo* studies reinforced these promising results, showing a significant reduction in bacterial growth (90%), diminished inflammation, improved tissue integration and accelerated wound healing over 14 days. However, this study has several limitations that must be considered. While *in vivo* studies were conducted, the implantation of the cement sample was limited to the subcutaneous tissue, which may not fully replicate the conditions in bone tissue, where the material is intended to be used clinically. Further studies should focus on implanting nanocomposite bone cement directly into bone tissue to evaluate its performance under more relevant biological conditions. Additionally, while the *in vitro* results and short-term *in vivo* studies are promising, the long-term efficacy, particularly the sustained antimicrobial effects and potential cytotoxicity, has not been fully evaluated. The complex process of incorporating AgNP@CDs into the PMMA matrix also requires further optimization to ensure consistent uniformity, scalability and cost-effectiveness for clinical applications. These findings demonstrate the potential of PMMA@2Ag-CDs as a biocompatible and antimicrobial alternative to traditional bone cement, offering an innovative solution for infection control and tissue regeneration in orthopedic and wound healing applications.

## Supplementary Material

rbaf086_Supplementary_Data
