# Supplementary material for: PMMA bone cement with AgNP@CDs nanocomposite for infection control and inflammation mitigation
Source: Regen Biomater. 2025 Aug 14;12:rbaf086. doi: 10.1093/rb/rbaf086 (PMC12448944; doi:10.1093/rb/rbaf086)
Supplement: rbaf086_Supplementary_Data [file rbaf086_supplementary_data.docx]

**PMMA bone cement with AgNP@CDs nanocomposite for infection control and inflammation mitigation” in the revised manuscript**

**Ihsan** **Ullah^1,2,3^, Jian** **Ju^3*^, Yapei Song^4^, Siyi** **Chen^5^, Mengshi** **Chen^6^, Siran** **Wang^3^, Wenzhen** **Zhang^7^, Wenhui** **Chen^8^, Zhifeng** **You^3, 9^, Huaqiong** **Li^1, 3^*, Feng** **Wen^4, 5, 6^*, Wei Zuo^1^**

^1^Joint Research Centre on Medicine, The Affiliated Xiangshan Hospital of Wenzhou Medical University, Ningbo, Zhejiang, 315700, China

^2^College of Chemical Engineering, Fuzhou University, Fuzhou, Fujian 350116, China

^3^Zhejiang Engineering Research Centre for Tissue Repair Materials, Wenzhou Institute, University of Chinese Academy of Sciences, Wenzhou, Zhejiang, 325001, China

^4^Postgraduate training base Alliance of Wenzhou Medical University, Wenzhou, Zhejiang, 325000, China,

^5^Zhejiang Top-medical Medical Dressing Co. Ltd, Wenzhou, Zhejiang, 325025, China

^6^Key Laboratory of Biomaterials and Biofabrication for Tissue Engineering, Gannan Medical University, Ganzhou, Jiangxi, 341000, China

^7^The Second Affiliated Hospital and Yuying Children's Hospital of Wenzhou Medical University, Wenzhou, Zhejiang, 325035, China

^8^Yuhuan People's Hospital, Taizhou, Zhejiang, 317600, China

^9^Faculty of Biomedical Engineering, Shenzhen University of Advanced Technology, Shenzhen, 518107, China

* **Correspondence address E-mail**: jujian@ucas.ac.cn (J.J); lihq@ucas.ac.cn (H.L); wenfeng@ucas.ac.cn (F.W)

**Table S1.** Summary of polymethylmethacrylate bone cement additives.

| **Additives** | **Functions** | **Side effects** | **Examples** |
| --- | --- | --- | --- |
| Antibiotics | Preventing or treating infection though high local concentration. | Reducing mechanical property, prompting antibiotic resistance potential. | Gentamicin, vancomycin, tobramycin. |
| Radiopacifiers | Improving visibility under X-ray/fluoroscopy for precise location during operation and long-term monitoring. | Inducing inflammation/osteolysis, weakening mechanical property. | Barium sulfate, zirconium dioxide. |
| Modifiers to decrease polymerization temperature | Reducing the curing temperature to prevent thermal necrosis of surrounding tissue. | Changing viscosity, setting time, and mechanical property. | Calcium carbonate, bioglass, hydroxyapatite. |
| Biocompatibility & bioactivity enhancers | Ameliorating bone-cement interface bonding, facilitating osteointegration. | Affecting mechanical strength. | Hydroxyapatite, strontium, collagen. |
| Mechanical property modifiers | Modulating fatigue property, fracture toughness, or elasticity. | Challenging even dispersion, Introducing weak interfaces. | Carbon fibers, polyethylene fibers. |
| Drugs (except antibiotics) | Deliver therapeutic agents locally. | Affecting mechanical strength. | Zoledronate, bone morphogenetic protein. |


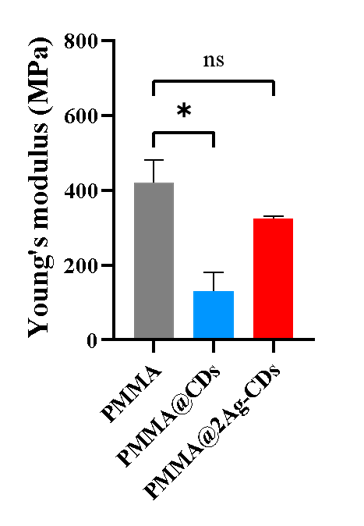


**Figure S1.** The Young’s modulus of bone cement and bone cement composite scaffold.


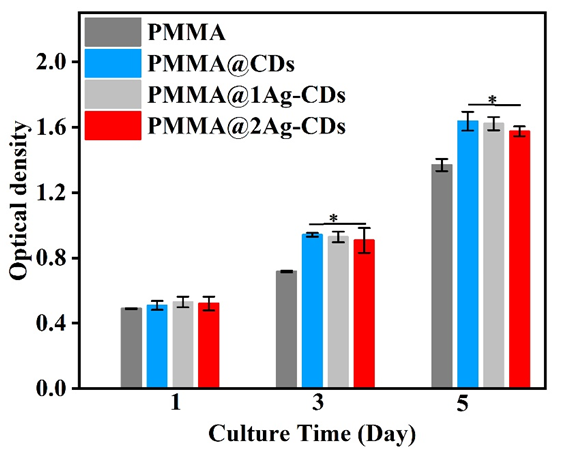


**Figure S2.** Cell proliferation of MC3T3 cells after 1, 3, and 5 days of culture on various PMMA samples.


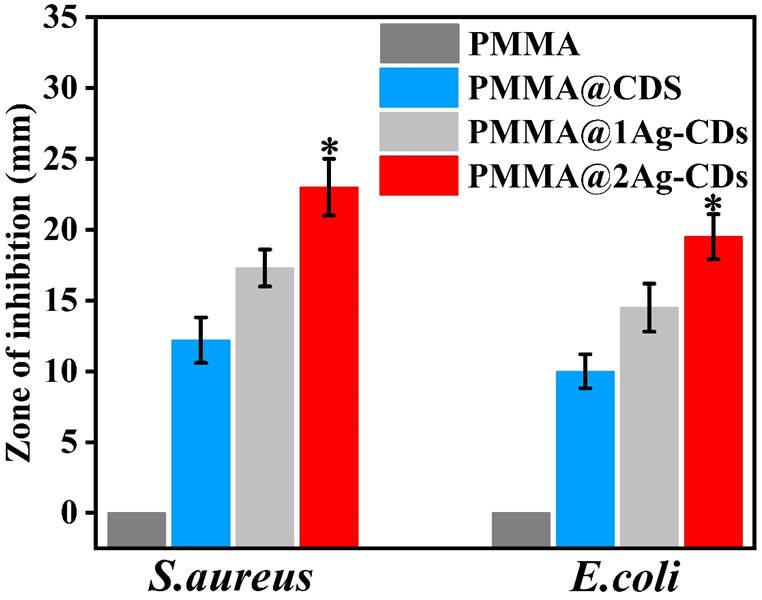


**Figure S3.** The inhibition zone of PMMA and its various composite against S.aureus and E.coli.





**Figure S4.** Fibrous layer thickness after 7 and 14 days of implantation.
